# MNBC: a multithreaded Minimizer-based Naïve Bayes Classifier for improved metagenomic sequence classification

**DOI:** 10.1093/bioinformatics/btae601

**Published:** 2024-10-10

**Authors:** Ruipeng Lu, Tim Dumonceaux, Muhammad Anzar, Athanasios Zovoilis, Kym Antonation, Dillon Barker, Cindi Corbett, Celine Nadon, James Robertson, Shannon H C Eagle, Oliver Lung, Josip Rudar, Om Surujballi, Chad Laing

**Affiliations:** National Centre for Animal Disease, Canadian Food Inspection Agency, Lethbridge County, AB, T1J 5R7, Canada; Saskatoon Research and Development Centre, Agriculture and Agri-Food Canada, Saskatoon, SK, S7N 0X2, Canada; Saskatoon Research and Development Centre, Agriculture and Agri-Food Canada, Saskatoon, SK, S7N 0X2, Canada; Department of Biochemistry and Medical Genetics, University of Manitoba, Winnipeg, MB, R3E 0J9, Canada; National Microbiology Laboratory at Winnipeg, Public Health Agency of Canada, Winnipeg, MB, R3E 3M4, Canada; National Microbiology Laboratory at Winnipeg, Public Health Agency of Canada, Winnipeg, MB, R3E 3M4, Canada; National Microbiology Laboratory at Winnipeg, Public Health Agency of Canada, Winnipeg, MB, R3E 3M4, Canada; National Microbiology Laboratory at Winnipeg, Public Health Agency of Canada, Winnipeg, MB, R3E 3M4, Canada; National Microbiology Laboratory at Guelph, Public Health Agency of Canada, Guelph, ON, N1G 3W4, Canada; National Microbiology Laboratory at Guelph, Public Health Agency of Canada, Guelph, ON, N1G 3W4, Canada; National Centre for Foreign Animal Disease, Canadian Food Inspection Agency, Winnipeg, MB, R3E 3M4, Canada; National Centre for Foreign Animal Disease, Canadian Food Inspection Agency, Winnipeg, MB, R3E 3M4, Canada; Ottawa Animal Health Laboratory, Canadian Food Inspection Agency, Ottawa, ON, K2J 4S1, Canada; National Centre for Animal Disease, Canadian Food Inspection Agency, Lethbridge County, AB, T1J 5R7, Canada

## Abstract

**Motivation:**

State-of-the-art tools for classifying metagenomic sequencing reads provide both rapid and accurate options, although the combination of both in a single tool is a constantly improving area of research. The machine learning-based Naïve Bayes Classifier (NBC) approach provides a theoretical basis for accurate classification of all reads in a sample.

**Results:**

We developed the multithreaded Minimizer-based Naïve Bayes Classifier (MNBC) tool to improve the NBC approach by applying minimizers, as well as plurality voting for closely related classification scores. A standard reference- and test-sequence framework using simulated variable-length reads benchmarked MNBC with six other state-of-the-art tools: MetaMaps, Ganon, Kraken2, KrakenUniq, CLARK, and Centrifuge. We also applied MNBC to the “marine” and “strain-madness” short-read metagenomic datasets in the Critical Assessment of Metagenome Interpretation (CAMI) II challenge using a corresponding database from the time. MNBC efficiently identified reads from unknown microorganisms, and exhibited the highest species- and genus-level precision and recall on short reads, as well as the highest species-level precision on long reads. It also achieved the highest accuracy on the “strain-madness” dataset.

**Availability and implementation:**

MNBC is freely available at: https://github.com/ComputationalPathogens/MNBC.

## 1 Introduction

The ability to detect pathogenic microorganisms in animals, food, and the environment is critical to ensuring animal and human health. Historically, this has been done using standard microbiology techniques that rely on the isolation of individual organisms. While these laboratory-based methods are effective, they are time- and resource-intensive, often requiring days to isolate pure cultures and characterize the agents of interest. By contrast, culture-independent metagenomics enables the direct capture and sequencing of all nucleic acid materials present in a sample (Thomas *et al.* 2012); however, organisms present in low numbers may still be undetectable. A single run of the Oxford Nanopore Promethion can generate up to 200 gigabases (GB) per run, with read lengths averaging about 10 kilobases (kb), but in some cases reaching 4 megabases (Mb) (https://nanoporetech.com/products/sequence/promethion); the Illumina NextSeq 550 can generate up to 120 Gb per run at a consistent sequence length of 2 × 150 base pairs (bp) (https://www.illumina.com/systems/sequencing-platforms.html).

Due to the fact that metagenomes contain genome sequences of all organisms detected in the sample, the reads from individual isolates would ideally be able to be separated from one another. In reality, this is often not possible, and classification of individual reads to the species, or even higher taxonomic level is the best that can be accomplished (Menzel *et al.* 2016). Each read is assigned a taxon, and the reads from the same species can be assembled into a metagenome-assembled genome (MAG) (Menzel *et al.* 2016). Two categories of classification tools exist: sequence alignment-based and *k*-mer composition-based. Some alignment-based tools [MetaPhyler (Liu *et al.* 2011), MetaPhylAn4 (Blanco-Míguez *et al.* 2023), mOTUs (Ruscheweyh *et al.* 2021)] map reads to a database of marker genes by using general aligners [BLAST (McGinnis and Madden 2004), BowTie2 (Langmead and Salzberg 2012), BWA (Li and Durbin 2009), respectively]. The alignment-based Centrifuge (Kim *et al.* 2016) uses the Burrows-Wheeler transform (Burrows and Wheeler 1994) and Ferragina-Manzini index (Ferragina and Manzini 2000) to index the merged reference genomes to accelerate aligning.

*K*-mers, which are sequence fragments of length *k*, are comparable to individual words that combine to form an article. They are used as the basic elements for classification in many tools. CLARK (Ounit *et al.* 2015) makes separate classifications at each taxonomic level by using only discriminative *k*-mers. Phymm (Brady and Salzberg 2009) uses Interpolated Markov Models trained with variable-length *k*-mers to construct nucleotide probability distributions of reference genomes. Mash (Ondov *et al.* 2016) produces MinHash (Broder 1997) bottom sketches from canonical *k*-mers and computes Jaccard index-based distances. Minimizers (Roberts *et al.* 2004), which are representative *k*-mers, are often used to reduce storage and speed up sequence comparison. Kraken (Wood and Salzberg 2014) builds a minimizer-indexed reference database mapping each *k*-mer to the lowest common ancestor (LCA) taxon of all genomes containing it, then the taxa associated with *k*-mers in a query read form a pruned subtree whose root is the prediction. KrakenUniq (Breitwieser *et al.* 2018) additionally counts unique *k*-mers for each taxon using a probabilistic cardinality estimator HyperLogLog (Flajolet *et al.* 2007, Heule *et al.* 2013) to reduce false positives. Kraken2 (Wood *et al.* 2019) further adopts a probabilistic, compact hash table directly mapping minimizers to LCAs to compress the database and run faster. Ganon (Piro *et al.* 2020) uses Interleaved Bloom Filters (Dadi *et al.* 2018) as the database to store minimizers and the q-gram lemma (Jokinen and Ukkonen 1991, Reinert *et al.* 2015) to classify reads. Long reads-oriented MetaMaps (Dilthey *et al.* 2019) uses a minimizer-based approximate mapping strategy to produce a list of candidate locations and an expectation–maximization (EM) algorithm (Dempster *et al.* 1977) to disambiguate.

*K*-mers were also used as features in machine learning-based tools. The single-threaded Naïve Bayes Classifier (NBC) (Rosen *et al.* 2008) applies Bayes’ Theorem that assumes independence of *k*-mers. The proof-of-concept NBC++ (Zhao *et al.* 2020) adds multithreading capability and optimizes memory and the number of cores via a smart loading scheme. MetaVW (Vervier *et al.* 2018) uses the squared loss function in the Vowpal Wabbit library (Langford *et al.* 2007, Agarwal *et al.* 2014) to train a separate classifier with random sequence fragments at each taxonomic level. MT-MAG (Li *et al.* 2023) builds a quadratic Support Vector Machine (QSVM) model (Cortes and Vapnik 1995) at each node of the taxonomy tree and classifies hierarchically from top to bottom, outperforming the deep learning-based DeepMicrobes (Liang *et al.* 2020) at the species level.

In this study, we developed a multithreaded Minimizer-based Naïve Bayes Classifier (MNBC) for improved metagenomic sequence classification, which includes four specific improvements to NBC: (i) the use of unique minimizers rather than all *k*-mers; (ii) binary presence/absence of a minimizer rather than its frequency; (iii) the use of a minimum cutoff for the ratio of shared read & genome minimizers to all read minimizers; and (iv) the use of the plurality rule in selecting read classification. To assess its performance, MNBC was benchmarked against six other state-of-the-art metagenomics read classification tools. It efficiently identified unknown reads with reasonable runtime and memory requirements, and outperformed other tools, with the exception of MetaMaps with regard to recall on long reads, in terms of species-level precision and recall across both short and long reads.

## 2 Materials and methods

### 2.1 Implementation of the NBC tool

The NBC tool (Rosen *et al.* 2008) uses the classic Naïve Bayes classifier to assign sequencing reads to categories as follows. Suppose that the reference database consists of S genomes {$G_{1}$, $G_{2}$,…,$\;G_{S}$}. The query read sequence R = [$m_{1}$, $m_{1}^{\prime }$, $m_{2}$, $m_{2}^{\prime }$,…,$\;m_{N}$, $m_{N}^{\prime }$] contains 2N*k*-mers; i.e. $m_{j}$ and $m_{j}^{\prime }$ are the two complement *k*-mers in the two strands at the position $j\;\left(1\leq j\leq N\right)$. $P\left(G_{i}|R\right)$ is the posterior probability of R originating from $G_{i}$ (1≤i≤S), and the Naïve Bayes classifier predicts R to originate from the genome $G_{\eta }$ with the greatest posterior probability where

(1) $$\eta =\arg\underset{i}{\max}P\left(G_{i}|R\right),\;1\leq i\leq S$$

where $P\left(G_{i}|R\right)$ is calculated based on Bayes’ Theorem as

(2) $$P\left(G_{i}|R\right)=\frac{P\left(R|G_{i}\right)P\left(G_{i}\right)}{P\left(R\right)}$$

$P\left(R\right)$ (i.e. the unconditional probability of observing R) is constant across all genomes so that it can be omitted. $P\left(G_{i}\right)$ (i.e. the prior probability of observing $G_{i}$) depends on the composition of a specific sample; without such prior knowledge all genomes can be assumed to be equally likely so that it can also be omitted. Since the Naïve Bayes classifier assumes that the features (i.e. *k*-mers in this case) are independent of one another, the conditional probability $P\left(R|G_{i}\right)$ can be calculated based on the product rule (Feller 1991) as

(3) $$P\left(G_{i}|R\right)\propto \;P\left(R|G_{i}\right)=\prod_{j=1}^{N}P\left(m_{j}|G_{i}\right)P\left(m_{j}^{\prime }|G_{i}\right)$$

$P\left(m_{j}|G_{i}\right)$ is the probability of observing $m_{j}$ in $G_{i}$, which is calculated as the number of occurrences of $m_{j}$ in $G_{i}$ [i.e. $\mathrm{count}\left(m_{j},G_{i}\right)$] divided by the total number of *k*-mers in $G_{i}$ [i.e. $\mathrm{count}\left(G_{i}\right)$]. To prevent precision errors that may be caused by multiplication of many small numbers in the case of a large N, a logarithm was introduced into Equation (3) to compute the score of $G_{i}$:

(4) $$\log P\left(R|G_{i}\right)=\sum_{j=1}^{N}\left(\log\frac{\mathrm{count}\left(m_{j},G_{i}\right)}{\mathrm{count}\left(G_{i}\right)}+\log\frac{\mathrm{count}\left(m_{j}^{\prime },G_{i}\right)}{\mathrm{count}\left(G_{i}\right)}\right)$$

Thus NBC predicts R to originate from the genome $G_{\eta }$ with the greatest score, where

(5) $$\eta =\arg\underset{i}{\max}\log P\left(R|G_{i}\right),\;1\leq i\leq S$$

### 2.2 Reference database building in MNBC

As indicated by Equation (4), the NBC tool builds the reference database by counting the number of occurrences of each present *k*-mer for each genome. To improve the runtime of the program, MNBC instead obtains unique minimizers in each genome. One minimizer is chosen from each window of length 2*k*-1 (i.e. *k* consecutive *k*-mers) as follows. At each position, the lexicographically smaller one of the two complement *k*-mers on the two strands is used as the canonical *k*-mer, following which the minimizer is the lexicographically smallest among all *k* canonical *k*-mers in the window. Thus the minimizer is a representative *k*-mer of the window, and adjacent windows often have the same minimizer, which reduces the size of the NBC database. Additionally, at either end of the genome sequence, *k*-1 minimizers are also chosen in the same way from *k*-1 windows that are anchored to this end and respectively consist of 1,2,…,*k*-1 consecutive *k*-mers.

MNBC hashes each minimizer $z=\left[b_{k-1}\ldots b_{1}b_{0}\right]$ to a number as follows to reduce its memory footprint. $b_{i}$ is the nucleotide base at the position $i\;\left(0\leq i\leq k-1\right)$, which is first mapped to a number:

(6) mapbi=0, if bi=A1, if bi=C2, if bi=G3, if bi=T

The hash number of z is computed as:

(7) $$\mathrm{hash}\left(z\right)=\;\sum_{i=0}^{k-1}\mathrm{map}\left(b_{i}\right)\cdot 4^{i}$$

Consistent with NBC which performed the best using 15-mers, *k* was also set to 15 in this study, which allows storage of $\mathrm{hash}\left(z\right)$ in a 4-byte integer type. To allow parallel processing during database building, the MNBC database consists of independent index files, each of which stores the total number of *k*-mers and hash numbers of unique minimizers in a reference genome. This also enables easy plug-in updates to the database; i.e. a genome can be included in or excluded from the database simply by adding or removing its index file.

### 2.3 Query read classification in MNBC

The original NBC tool, due to the sheer size of its database, loads all query reads into memory, then sequentially computes the scores of each reference genome [Equation (4)]. Thanks to using minimizers instead of all *k*-mers, MNBC is able to keep the entire database in memory and classify multiple reads in parallel.

Given a query read R, MNBC first computes hash numbers of its unique minimizers. To identify reads from unknown microorganisms, MNBC introduces a minimum cutoff μ on the ratio of shared read & genome minimizers to all read minimizers. A genome with a ratio smaller than μ will not be considered as a match for the read and thus be rejected. In the special case of μ=0, a genome sharing no minimizer with the read will be rejected. If all genomes in the database are rejected, the read will be labeled as unclassified.

To improve the classification speed of NBC, MNBC simplifies Equation (4) by ignoring the exact numbers of occurrences of the read minimizers in the reference genome; i.e. for each present minimizer, MNBC uses 1 as its number of occurrence. If a read minimizer is absent in the genome, the logarithm in Equation (4) will approach negative infinity; to account for this, MNBC uses a penalty parameter φ to replace the logarithm. Assuming that R contains U unique minimizers {$z_{1},\;z_{2},\ldots ,\;z_{U}$}, MNBC uses the simplified Equation (8) to compute the score of the genome $G_{i}$:

(8) log⁡PRGi=∑j=1Ulog⁡1countGi, if zj is present in Giφ, if zj is absent in Gi

To improve on the performance of the original NBC algorithm, which classifies a read into the category with the greatest score [Equation (5)], despite occasions when the scores of many categories differ only slightly, MNBC considers multiple top scores, by applying a maximum threshold θ on the difference between adjacent scores. When the scores of reference genomes are sorted in descending order, MNBC sequentially computes the difference between each score and the previous one; if it is not >θ, the genomes with the score are added as candidates. This process stops when the first difference between sorted scores exceeds θ. Thus these candidate reference genomes with top scores are the best matches for R. Based on likelihood maximization, MNBC predicts R to originate from the species with the most candidate genomes, and randomly chooses one if multiple such species are present. Other more complex approaches to disambiguate multiple candidate species were also tested, such as choosing the one with the greatest score or average score, but they underperformed random choice besides slowing down classification speed. Taking into account only the highest score in NBC is a special case where θ is set to 0.

### 2.4 Assessment of the performance and practicality of MNBC

**Figure 1.**
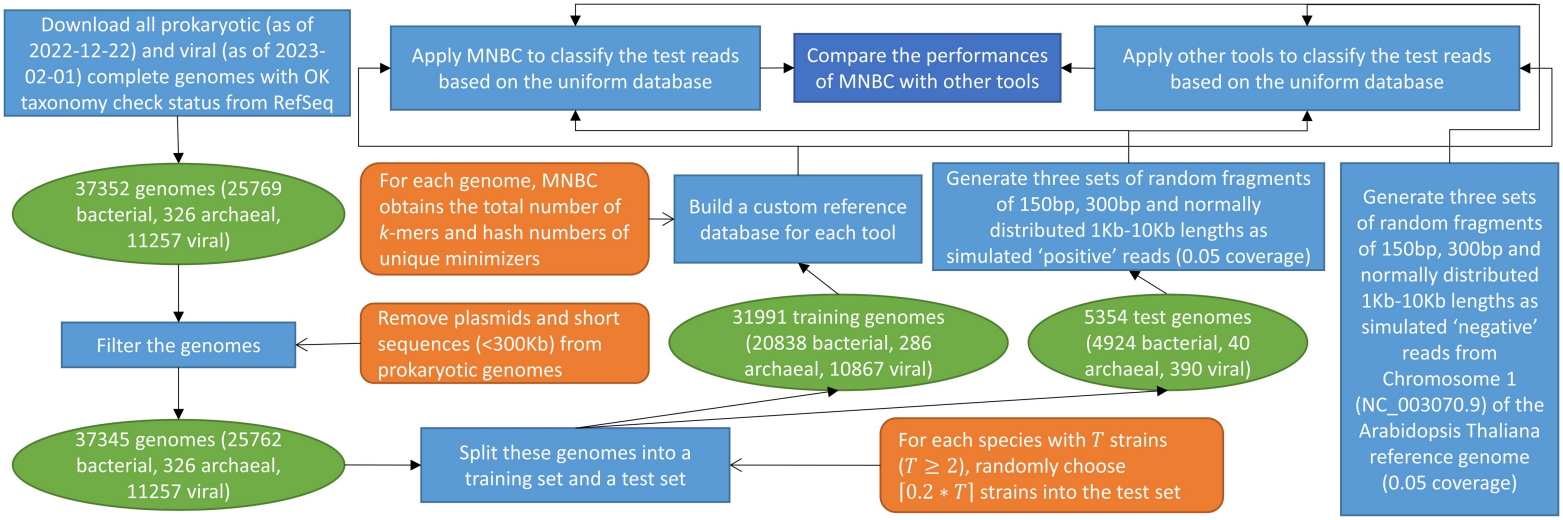
The standard framework to benchmark read classifiers. Prokaryotic and viral complete genomes with OK taxonomy check status were downloaded from the NCBI RefSeq collection. Plasmids and short sequences were removed from prokaryotic genomes as a filtering step. For each species with at least two strains, 20% of its strains were randomly picked into a test set. All remaining genomes were put into a training set. Every tool built a custom reference database from the training set. Genomes in the test set were used to respectively generate three sets of random sequence fragments of 150 bp, 300 bp and normally distributed 1–10 kb lengths as simulated positive reads. On the other hand, Chromosome 1 of the RefSeq reference genome of the *Arabidopsis thaliana* plant was used to respectively generate three sets of random sequence fragments of corresponding lengths as simulated negative reads. All tools classified these test reads based on the uniform database using the same hardware, then their performances were compared.

To assess the performance of MNBC, we developed a standard framework to benchmark it against six other state-of-the-art tools (MetaMaps v0.1, Ganon v1.8.0, Kraken2 v2.1.2, CLARK v1.2.6.1, Centrifuge v1.0.4, KrakenUniq v0.5.7) using simulated sequencing reads (Fig. 1). First we downloaded all prokaryotic complete genomes as of 22 December 2022 and viral complete genomes as of 1 February 2023 with OK taxonomy check status from the NCBI RefSeq collection. The prokaryotic genomes were filtered by removing plasmids and sequences shorter than 300 kb. For each species with T strains (T≥2), $\left\lceil 0.2*T\right\rceil$ strains were randomly chosen into a test set. The remaining training genomes were used by every tool to build a custom reference database. To simulate “positive” reads from known microorganisms produced by different sequencing platforms (NextSeq, MiSeq and Nanopore), the test genomes were used to respectively generate 6 562 565 random sequence fragments of 150 bp length, 3 282 728 fragments of 300 bp length and 181912 fragments of normally distributed 1–10 kb lengths based on 0.05 coverage. To simulate “negative” reads from unknown microorganisms that do not exist in the database, Chromosome 1 (NC_003070.9) of the RefSeq reference genome of the *Arabidopsis thaliana* plant was used to generate 10143, 5072, and 277 random sequence fragments of corresponding lengths based on 0.05 coverage. Thus the framework produced a uniform database, three metagenomic sets of positive reads and three negative read sets used by all tools. Comparison fairness was also ensured by running all tools using 100 cores of the same computer with an AMD Ryzen Threadripper 3990X CPU and 256 gigabytes (GB) memory.

On negative reads, accuracy was defined as the percentage of reads that were left unclassified. On positive reads, precision at each taxonomic level = $\frac{\#\;\mathrm{reads}\;\mathrm{classified}\;\mathrm{correctly}\;\mathrm{to}\;\mathrm{this}\;\mathrm{level}}{\#\;\mathrm{reads}\;\mathrm{classified}}$ and recall (or accuracy) = $\frac{\#\;\mathrm{reads}\;\mathrm{classified}\;\mathrm{correctly}\;\mathrm{to}\;\mathrm{this}\;\mathrm{level}}{\#\;\mathrm{reads}}$.

To further assess the practicality of MNBC on real-world metagenomic sequencing runs, we applied it to the strain-madness and marine datasets (Fritz *et al.* 2020) in the CAMI II challenge (Meyer *et al.* 2022). The CAMI II challenge, which was open from 16 January 2019 to 25 October 2019, benchmarked taxonomic classification tools on realistic and complex metagenomic datasets with computationally generated long- and short-read sequences. Since the majority of the participating tools only analyzed short reads, we also used MNBC to classify the same sequences. The strain-madness dataset has very high strain diversity, and each of its 100 samples contains 2 GB of short (150 bp) paired-end reads. The ten samples in the marine dataset were created from a deep-sea environment, each containing 5 GB of short paired-end Illumina reads. To ensure that the results of MNBC were comparable to the other participating tools, we used the RefSeq database as of 15 October 2019, containing all 16 864 prokaryotic and viral complete genomes and chromosomes with OK and inconclusive taxonomy check statuses.

## 3 Results

### 3.1 MNBC performance on the simulated test reads

**Figure 2.**
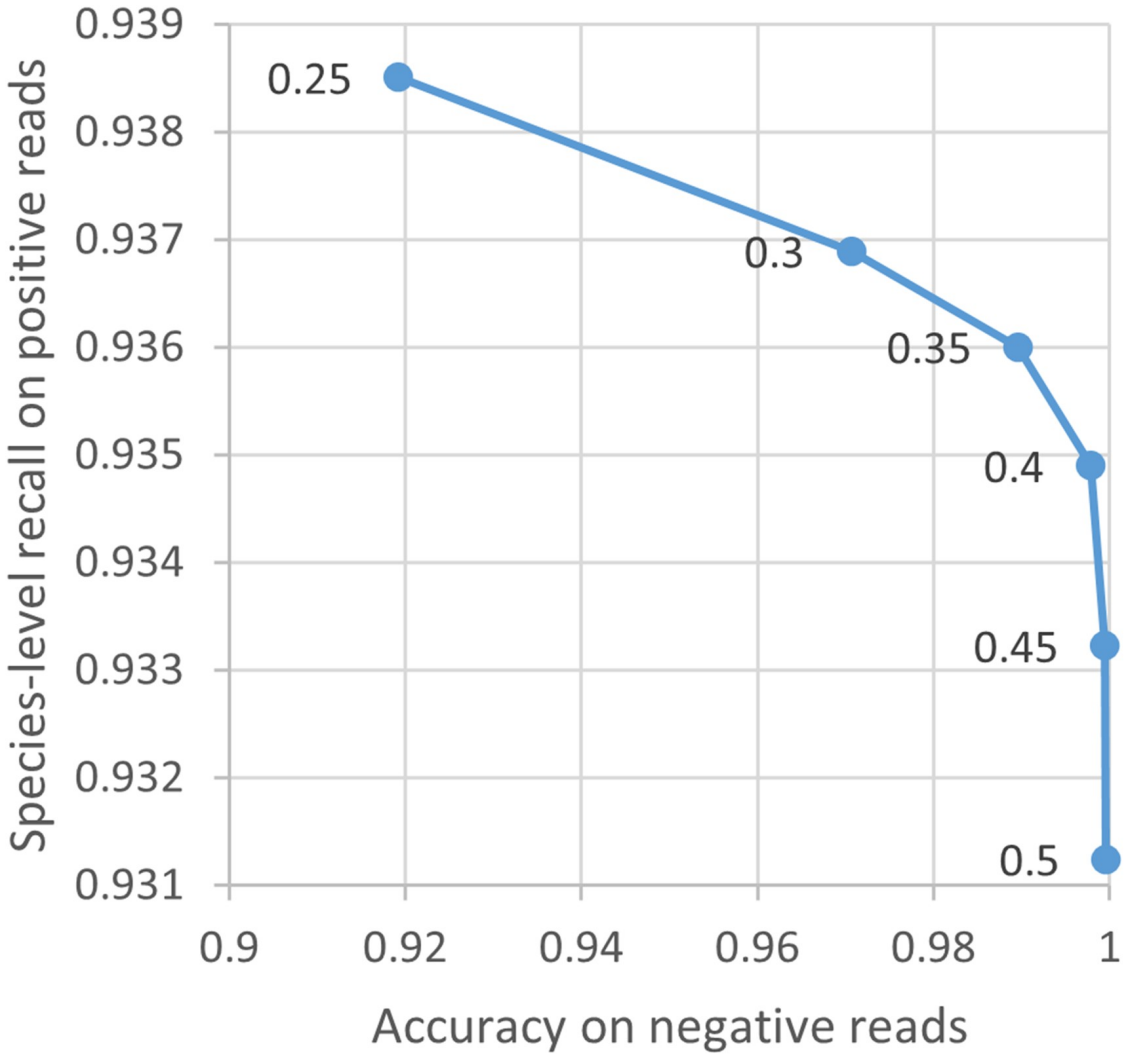
Effect of µ on the classification behavior of MNBC. Each blue point represents an experiment on the MiSeq test reads using a different µ value indicated by the adjacent number. The raw numbers are provided in Supplementary Table S1.

To determine an optimal value of the μ parameter, we experimented on the MiSeq test reads to examine its effects on the classification behavior of MNBC. A larger μ value is related to a higher accuracy on the negative reads and a lower species-level recall on the positive reads (Fig. 2). A threshold of 0.35 was subsequently used to achieve a good balance, meaning that a genome is rejected if it contains <35% of the read minimizers.

The results of benchmarking MNBC with six other state-of-the-art tools on the NextSeq, MiSeq and Nanopore test reads are respectively shown in Tables 1–3. We adjusted the parameters of these tools in an attempt to obtain the highest possible performances.

**Table 1.** Performances of the seven read classifiers on the NextSeq test reads.^a^

Tool	Accuracy on negative reads (%)	On positive reads									
		Precision/recall (or accuracy) (%)							Percentage of unclassified reads (%)	Runtime (hh:mm:ss)	Memory footprint (GB)
		Species	Genus	Family	Order	Class	Phylum	Domain			
MNBC	95.37	94.24/92.63	98.71/97.03	99.71/98.01	99.80/98.10	99.90/98.19	99.94/98.23	99.99/98.28	1.71	02:36:52	182.09
Ganon	99.89	90.60/89.17	96.18/94.66	99.81/98.24	99.90/98.32	99.96/98.38	99.98/98.41	99.99/98.41	1.58	00:50:22	87.39
Centrifuge	95.95	72.29/52.31	96.74/70.00	99.04/71.66	99.27/71.82	99.41/71.93	99.58/72.05	99.66/72.11	27.65	00:38:12	38.99
Kraken2	99.86	54.81/54.12	89.10/87.98	98.58/97.34	98.97/97.73	99.22/97.97	99.36/98.10	99.49/98.24	1.26	00:00:14	31.67
KrakenUniq	99.64	54.66/54.00	89.08/88.00	98.64/97.45	99.01/97.81	99.24/98.04	99.37/98.16	99.48/98.28	1.21	00:03:08	1.24
CLARK	99.68	54.61/53.86	89.21/87.98	99.09/97.72	99.63/98.26	99.86/98.49	99.99/98.61	99.99/98.61	1.38	00:07:52	99.02
MetaMaps	0	NA									

a Positive reads were from known microorganisms that exist in the reference database, and negative *Arabidopsis thaliana* reads simulated reads from unknown microorganisms. All tools were run with the same hardware against the uniform database, and listed in the descending order of species-level precision on positive reads. MNBC respectively used 0.35, −2000, and 1500 as the values of the μ, φ, and θ parameters. 200 GB was set as its maximum heap size in Java 17.0.4.1 and not the minimal required memory amount. Ganon used the “—binning” parameter based on the database with 0.001 false positive rate. Since CLARK makes classifications separately on different taxonomic levels, its species-level runtime and memory footprint are indicated. Default parameter values were used for all other tools. MetaMaps was specifically designed for long reads, and was not run on the positive reads due to its zero accuracy on the negative reads and slow speed. The raw numbers are provided in Supplementary Table S2.

**Table 2.** Performances of the seven read classifiers on the MiSeq test reads.^a^

Tool	Accuracy on negative reads (%)	On positive reads									
		Precision/recall (or accuracy) (%)							Percentage of unclassified reads (%)	Runtime (hh:mm:ss)	Memory footprint (GB)
		Species	Genus	Family	Order	Class	Phylum	Domain			
MNBC	98.96	95.09/93.60	99.06/97.51	99.82/98.25	99.89/98.32	99.95/98.38	99.97/98.41	99.99/98.42	1.57	01:59:15	196.34
Ganon	99.98	93.86/92.46	98.87/97.40	99.84/98.35	99.91/98.42	99.96/98.47	99.98/98.50	99.99/98.50	1.49	00:30:22	83.36
Centrifuge	92.11	76.64/60.32	96.56/76.01	99.14/78.03	99.33/78.18	99.47/78.29	99.62/78.41	99.72/78.49	21.29	00:15:31	38.96
Kraken2	99.68	62.85/62.28	91.01/90.19	98.96/98.07	99.27/98.38	99.44/98.54	99.55/98.65	99.64/98.74	0.90	00:00:13	31.58
KrakenUniq	99.45	61.90/61.30	90.71/89.84	98.98/98.02	99.27/98.32	99.43/98.48	99.54/98.59	99.62/98.66	0.96	00:02:11	1.21
CLARK	99.51	61.89/61.24	90.80/89.84	99.31/98.26	99.72/98.66	99.88/98.82	99.98/98.93	99.98/98.93	1.06	00:09:12	99.34
MetaMaps	0	NA									

a The same parameter settings as on the NextSeq test reads were used. The raw numbers are provided in Supplementary Table S3.

**Table 3.** Performances of the seven read classifiers on the Nanopore test reads.^a^

Tool	Accuracy on negative reads (%)	On positive reads									
		Precision/recall (or accuracy) (%)							Percentage of unclassified reads (%)	Runtime (hh:mm:ss)	Memory footprint (GB)
		Species	Genus	Family	Order	Class	Phylum	Domain			
MNBC	100	96.15/94.89	99.44/98.14	99.84/98.53	99.88/98.58	99.91/98.60	99.93/98.62	99.93/98.62	1.31	01:46:06	201.64
Ganon	100	95.94/94.80	99.48/98.30	99.89/98.71	99.94/98.75	99.96/98.78	99.98/98.80	99.98/98.80	1.18	00:03:59	83.53
MetaMaps	100	95.56/95.16	99.36/98.94	99.83/99.41	99.89/99.47	99.93/99.51	99.95/99.53	99.95/99.53	0.41	07:58:30	235.80
Kraken2	80.51	90.03/89.96	98.06/97.99	99.68/99.61	99.79/99.71	99.86/99.79	99.92/99.84	99.96/99.88	0.07	00:00:20	31.49
KrakenUniq	84.84	84.46/84.33	96.65/96.49	99.66/99.50	99.76/99.59	99.83/99.66	99.89/99.72	99.91/99.74	0.16	00:02:00	1.11
CLARK	85.92	84.32/84.18	96.68/96.51	99.74/99.57	99.86/99.69	99.92/99.75	99.98/99.81	99.98/99.81	0.17	00:17:47	98.81
Centrifuge	27.44	87.94/83.56	97.98/93.10	99.53/94.57	99.64/94.68	99.74/94.77	99.82/94.85	99.91/94.93	4.98	00:00:59	37.20

a The same parameter settings as on the NextSeq test reads were used. MetaMaps used 200 GB as the value of the “maxmemory” parameter in the mapping step. Centrifuge occurs last due to its underperforming accuracy on the negative reads. The raw numbers are provided in Supplementary Table S4.

On the NextSeq and MiSeq negative reads, four tools (Ganon, Kraken2, KrakenUniq and Centrifuge) correctly recognized over 99% of them, followed by MNBC and Centrifuge (Tables 1 and 2). MetaMaps could not recognize any (Tables 1 and 2). On the positive reads, at the expense of runtime and memory MNBC exhibited the highest precision and recall at the species and genus levels, exceeded by Ganon at higher levels (Tables 1 and 2). Centrifuge, Kraken2, KrakenUniq and CLARK exhibited similar species-level results, except a higher precision of Centrifuge due to more unclassified reads (Tables 1 and 2). At higher levels, all tools exhibited almost perfect results, except a lower recall of Centrifuge (Tables 1 and 2). Kraken2 was the fastest tool (Tables 1 and 2).

On the Nanopore negative reads, three tools (MNBC, Ganon and MetaMaps) correctly recognized all of them, followed by Kraken2, KrakenUniq and CLARK (Table 3). Centrifuge only recognized over a quarter (Table 3). On the positive reads, at the species level, MNBC and MetaMaps, respectively, exhibited the highest precision and recall (Table 3). Similarly, MNBC had a higher precision and recall than Ganon at the expense of runtime and memory, exceeded by Ganon at higher levels (Table 3). Kraken2 visibly exceeded Centrifuge, KrakenUniq and CLARK (Table 3). At higher levels, all tools also exhibited almost perfect results, except a lower recall of Centrifuge due to more unclassified reads (Table 3). Kraken2 and MetaMaps were, respectively, the fastest and slowest tools (Table 3).

### 3.2 Misclassified reads

**Figure 3.**
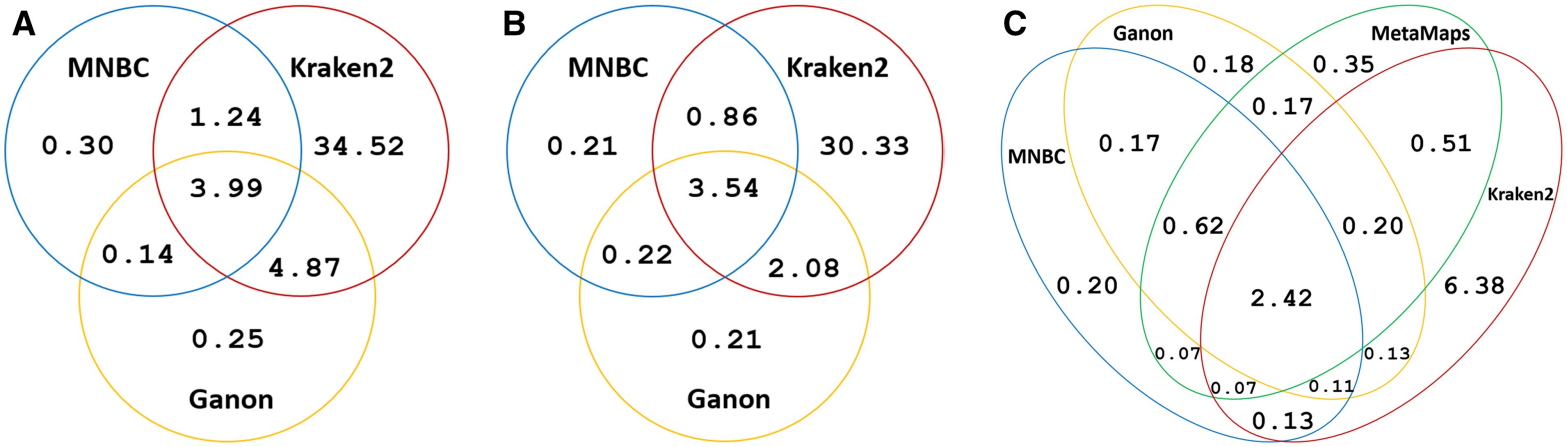
Percentages (%) of positive reads misclassified by MNBC, Ganon, MetaMaps, and Kraken2 at the species level. (A) Percentages of NextSeq positive reads misclassified by MNBC, Ganon, and Kraken2. (B) Percentages of MiSeq positive reads misclassified by MNBC, Ganon, and Kraken2. (C) Percentages of Nanopore positive reads misclassified by the four tools. The area within each ellipse represents all reads misclassified by a tool. The intersection between two ellipses represents the reads commonly misclassified by both tools. Each tool correctly classified reads that all other tools misclassified; each tool also misclassified reads that all others correctly classified; many reads were misclassified by all tools. The raw numbers are provided in Supplementary Fig. S1.

To further understand the classification behavior of the various tools, we examined the specific test reads misclassified at the species level by MNBC, Kraken2, Ganon and MetaMaps. Every tool had its unique correctly classified and misclassified reads; i.e. for each tool there existed some reads which only it could correctly classify or only it misclassified (Fig. 3). For instance, all tools misclassified 2.42% of the Nanopore positive reads, while MNBC correctly classified 0.2% misclassified by all three other tools, and misclassified 0.2% correctly classified by all others (Fig. 3C).

### 3.3 MNBC performance on the CAMI II datasets

To evaluate the practicality of MNBC in realistic metagenomic sequencing, we simulated participation in the CAMI II challenge by classifying the short reads of the strain-madness and marine datasets, based on a RefSeq database that existed while the challenge was open.

**Figure 4.**
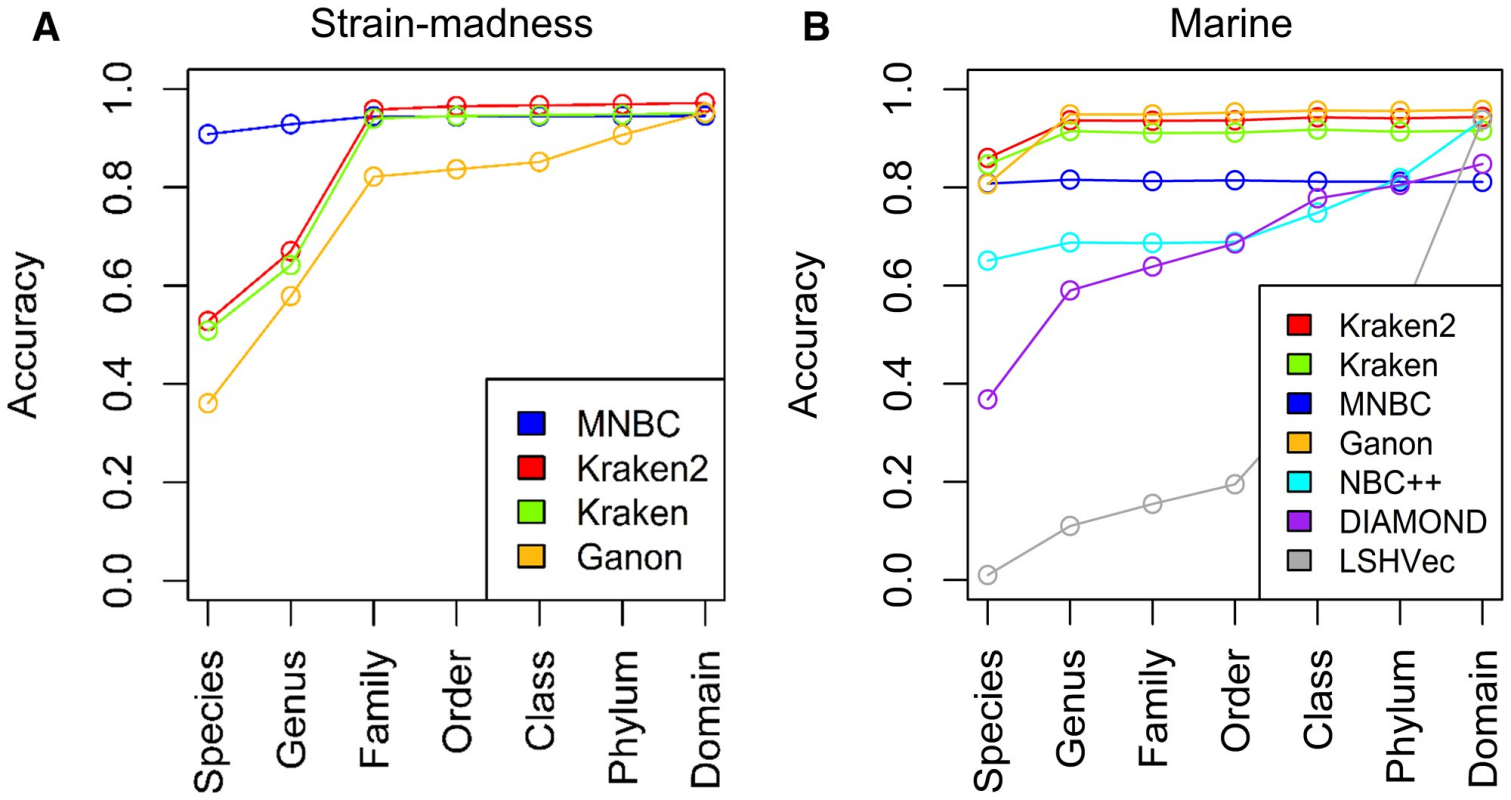
Performances of read classifiers on the short-read strain-madness and marine datasets of the CAMI II challenge. (A) Accuracies of MNBC and three participating tools on the strain-madness dataset. (B) Accuracies of MNBC and six participating tools on the marine dataset. MNBC respectively used 0.35, −2000, and 1500 as the values of the μ, φ, and θ parameters. The accuracies of all participating tools were directly obtained from Meyer *et al.* (2022), and the accuracies of MNBC were computed using the same length-based method. The tool versions participating in the challenge include Kraken2 v2.0.8, Kraken v0.10.5, Ganon v0.1.4 and DIAMOND v0.9.28, and each tool used its own reference database instead of a uniform one. The raw numbers are provided in Supplementary Table S5.

On the strain-madness dataset, which had high strain diversity, MNBC exhibited the highest accuracies at the species and genus levels, considerably outperforming the runner-up Kraken2 by about 38% and 26%, respectively (Fig. 4A).

On the marine dataset, the species-level top performers include Kraken2, Kraken, MNBC and Ganon (Fig. 4B). At higher levels, MNBC had lower accuracies than the other three tools. This is because MNBC had more unclassified reads on which the others instead gave incorrect species-level classifications, like on the simulated test reads.

## 4 Discussion

In this study, we took the NBC algorithm, and improved it by applying minimizers, a genome-rejection cutoff and the plurality rule, to create MNBC. The simplification of using minimizers reduces the size of the NBC reference database so that it can be held entirely in memory, which allows for much faster parallel read classification. The MNBC database is also fully customizable through the inclusion of any genomic sequence, and easily updatable through incremental additions that do not require the complete rebuilding of the database, as is the case with the six benchmarked tools. The minimum cutoff on the percentage of common read & genome minimizers allows rejection of dissimilar genomes and recognition of unknown reads. Plurality voting of candidate reference genomes from closely related top scores was found to also improve the accuracy of NBC.

The benchmarking results of the seven tools on the simulated reads indicated that at the species level, MNBC has the highest precision across short and long reads and the highest recall on short reads, while effectively recognizing unknown reads (Tables 1–3). MetaMaps and Centrifuge failed to recognize most unknown short and long reads, respectively (Tables 1–3). The longer the reads, the better the ability of MNBC and Ganon to recognize unknown reads, whereas the opposite is true for Kraken2, KrakenUniq and CLARK (Tables 1–3). No tool universally achieved both the highest classification power and fastest speed, and tool choice depends on the needs of the specific user. MNBC produced highly accurate classifications on the simulated read sets, while Kraken2 was extremely fast at the sacrifice of some accuracy. Ganon demonstrated a well-balanced approach, and MetaMaps works well on long reads despite being slow. The fact that every tool has its unique correctly classified and misclassified reads besides many common misclassifications suggests that every algorithm has its own advantages and no one is superior to all others in all cases.

The performance of MNBC is affected by the values of the μ, φ, and θ parameters. The range [0.3–0.45] of μ seems well-balanced between known and unknown reads (Fig. 2). φ must be smaller than $\log\left(\frac{1}{\mathrm{count}\left(G_{L}\right)}\right)$ in Equation (8) to distinguish present from absent read minimizers, where $G_{L}$ is the reference genome with the most *k*-mers (i.e. the longest genome). A smaller value of φ results in a larger score difference between two genomes with different numbers of read minimizers, thus θ should be set to a greater value to compensate for this. We found that higher absolute values of φ and θ gave better results on the simulated reads, arriving at defaults of −2000 and 1500, respectively.

There have been some previous works to compare read classifiers. NBC and Kraken2 were found to respectively excel at classification power and speed (Bazinet and Cummings 2012, Wood *et al.* 2019), and Ganon was a top performer at the species level (Seppey *et al.* 2020), which are consistent with our findings. The read-level classification powers of 11 tools were benchmarked on 35 simulated and biological metagenomic datasets (McIntyre *et al.* 2017), though the use of different reference databases complicated the explanation of their performance differences. A uniform database was used to benchmark species-level abundance profiling of 20 commonly used classifiers with simulated short reads (Ye *et al.* 2019), but it was in doubt whether the source taxa of the simulated reads still remained true, since the artificially introduced sequencing errors might make them actually closer to other reference genomes.

The marker-based tools (MetaPhyler, MetaPhylAn4, mOTUs) were excluded from the benchmarking experiment, since they are unable to classify reads originating outside the marker genes, and as such have lower performance (Ye *et al.* 2019). NBC++ generated over 1 terabyte (TB) of data before depleting hard drive space when we tried to build the uniform database. The single-threaded MetaVW took us over 12 days to train a 10-mer genus-level classifier of 1 coverage for the uniform database, and it was infeasible to train a 15-mer one due to over 1 TB memory usage. The single-threaded NBC and Phymm were also very slow taking over a week to finish classifying the test reads, and Mash significantly underperformed the benchmarked tools. MT-MAG is only suitable for local classification due to the sheer number of models needed to cover the entire taxonomy tree and its performance is upper bounded by the single top model. Nevertheless, the list of tools benchmarked in this study may not be exhaustive and more tools can be easily included if they allow custom database building.

In summary, we demonstrated that MNBC is a practical short- and long-read classifier for metagenomic sequencing with high species-level precision and recall and efficient recognition of unknown reads. As more reference genomes are deposited into the RefSeq collection, it is also expected to become increasingly more accurate due to plurality voting of candidate genomes from multiple top scores. Potential future work includes exploring further improvements to runtime, and applying the framework to other metagenomics classification tasks such as the identification of mobile elements.

## Supplementary Material

btae601_Supplementary_Data

## Data Availability

The data underlying this article are available in Zenodo, at https://dx.doi.org/10.5281/zenodo.10568965, https://dx.doi.org/10.5281/zenodo.10607025, and https://dx.doi.org/10.5281/zenodo.10601507.
